# Evidence-Based Approach of Biologic Therapy in Bronchial Asthma

**DOI:** 10.3390/jcm12134321

**Published:** 2023-06-27

**Authors:** Adnan Liaqat, Mathew Mason, Brian Foster, Grant Gregory, Avani Patel, Aisha Barlas, Sagar Kulkarni, Rafaela Basso, Pooja Patak, Hamza Liaqat, Muhammad Qureshi, Abdelrahman Shehata, Yousef Awad, Mina Ghaly, Qamar Gulzar, Walter Doty

**Affiliations:** 1 Pulmonary and Critical Care Medicine, McLaren Health/Michigan State University, Flint, MI 48532, USA; adnan.liaqat@mclaren.org; 2 Internal Medicine, Baylor College of Medicine, Houston, TX 77030, USA; biologictherapyasthma@gmail.com; 3 Pulmonary and Critical Care Medicine, Cleveland Clinic Florida, Weston, FL 33321, USA; bjfoster@southeasthealth.org; 4 Internal Medicine, Alabama College of Osteopathic Medicine, Dothan, AL 36303, USA; gregoryg@acom.edu; 5 Internal Medicine, Meharry Medical College, Nashville, TN 37208, USA; 6 Internal Medicine, Mercy Health, Rockford, IL 61114, USA; abarlas@mhemail.org; 7 Pulmonary and Critical Care Medicine, Southeast Health, Dothan, AL 36301, USA; skulkarni@southeasthealth.org (S.K.); rguintibasso@southeasthealth.org (R.B.); mqureshi@southeasthealth.org (M.Q.); abde.abde@gmail.com (A.S.); yousefmawad9@gmail.com (Y.A.); mmghaly@southeasthealth.org (M.G.); qamargulzar@gmail.com (Q.G.); walt.doty@gmail.com (W.D.); 8 Pulmonary and Critical Care Medicine, University of Missouri, Kansas City, MO 64110, USA; papatak@southeasthealth.org; 9 Internal Medicine, Wah Medical College, Wah Cantt 47040, Pakistan; 98hamzaliaqat@gmail.com

**Keywords:** asthma, omalizumab, reslizumab, benralizumab, mepolizumab, dupilumab, tezepelumab

## Abstract

The emergence of biologic agents in the treatment of bronchial asthma has a wide impact on improving quality of life, reducing morbidity, and overall health care utilization. These therapies usually work by targeting specific inflammatory pathways involving type 2 inflammation and are particularly effective in severe eosinophilic asthma. Various randomized controlled trials have shown their effectiveness by reducing exacerbation rates and decreasing required glucocorticoid dosages. One of the relatively newer agents, tezepelumab, targets thymic stromal lymphoprotein and has proven its efficacy in patients independent of asthma phenotype and serum biomarker levels. This article reviews the pathophysiologic mechanism behind biologic therapy and offers an evidence-based discussion related to the indication, benefits, and adverse effects of such therapies.

## 1. Introduction

The Global Initiative for Asthma (GINA) identifies asthma as a heterogeneous disease defined by chronic airway inflammation [1]. The airway inflammation associated with asthma is known to cause a broad spectrum of respiratory symptoms, including shortness of breath, cough, chest tightness, and wheezing. Known as a common condition, asthma affects over 334 million individuals globally [2]. There are several risk factors that contribute to the development and severity of asthma, including genetic predisposition, hormonal influences, sex, socioeconomic background, smoking, and obesity [2].

The hallmark of asthma pathophysiology is airway inflammation in response to a trigger, such as an environmental allergen, airway irritant, or exercise, which initiates a cascade of inflammation. Underlying airway inflammation is primarily driven by T-helper lymphocyte production of interleukins (IL), which in turn, leads to the recruitment of eosinophils, mast cells, basophils, neutrophils, monocytes, and macrophages [3]. Chronic airway inflammation contributes to airway remodeling, which is characterized by smooth muscle hypertrophy, increased basement membrane thickness, angiogenesis, mucus plugging, and airway narrowing [2].

Pharmacological treatment of asthma is separated into categories that include rescue therapy, controller medications, and for severe asthma, add-on therapies. The mainstay of treatment consists of reliever medications for breakthrough symptoms and controller medications to reduce airway inflammation. Most biologic agents are indicated for patients with severe asthma particularly with eosinophilic phenotype, who remain symptomatic with frequent exacerbations, despite treatment with high doses of inhaled corticosteroid (ICS) and long-acting beta-agonists (LABA) [1]. The targets for existing biologic agents include immunoglobulin E (IgE), IL-4, IL-5, IL-13, and thymic stromal lymphopoietin.

Our evolving understanding of the underlying pathophysiology of asthma has led to an ongoing revision of treatment guidelines and innovative treatment options. In our review, we discuss the current biologic therapies available for the treatment of bronchial asthma.

## 2. Pathophysiology of Asthma

Airway inflammation is an important pathophysiological marker in asthma. Inflammation can be classified as type 2 and non-type 2. Type 2 inflammation is the predominant phenotype in children and adults, which is often associated with atopy and characterized by an interleukin-mediated immune response to a trigger or allergen [4]. During allergen sensitization, antigen-presenting cells called dendritic cells activate the differentiation of naive T cells into type 2 helper (TH2) T cells. These TH2 cells are responsible for the production of IL-4, IL-5, IL-13, and granulocyte-macrophage colony-stimulating factor (GM-CSF), which promotes IgE synthesis, mast cell, eosinophil, and basophil recruitment [3,4]. Additionally, non-allergic triggers, such as infectious agents, air pollutants, and irritants can promote airway epithelial cell release of IL-25, IL-33, and thymic stromal lymphopoietin [4]. Non-type 2 inflammation may occur through multiple heterogeneous mechanisms that are not currently well understood. Non-type 2 inflammation is characterized by more neutrophilic or pauci-granulocytic infiltration and tends to be associated with obesity and smoking-related asthma [4]. It is acted upon by interleukins produced by TH1 and TH17 cells.

Biomarkers may provide some clinical data when evaluating patients with asthma. Their greatest utility is distinguishing type 2 from non-type 2 inflammatory states [3]. Type 2 inflammatory biomarkers include fractional exhaled nitric oxide (FeNO), blood eosinophils, sputum eosinophils, and periostin [5]. Due to differences in background cytokine regulation, these measurements are not always as effective in directing patient therapy; however, they are still utilized in clinical practice [6]. Microbiome and omics technologies (epigenomics, genomics, transcriptomics, and others) have been proposed as potential biomarkers, although these are still in the early phase of research [7].

## 3. Selecting Biologic Therapy in Bronchial Asthma

Current literature reviewing randomized controlled trials (RCTs) suggests that traditional non-biologic therapies are efficacious in improving outcomes of most patients with severe asthma [8]. GINA 2021 guidelines and the National Asthma Education and Prevention Program (NAEPP) recommend the use of inhaled corticosteroids or beta-agonists in the initial treatment of asthma [1,9]. If symptoms persist and asthma exacerbations remain uncontrolled, long-acting muscarinic antagonists may be employed in addition to the above therapies [1,9]. Until the development of the first biologic agents, such as omalizumab, in the early 2000s, oral corticosteroids were the last line of defense in the treatment of severe asthma. However, corticosteroid use was associated with many deleterious effects, such as adrenal insufficiency, Cushing syndrome, glaucoma, cataracts, iatrogenic diabetes mellitus, and osteoporosis [10]. In addition to such debilitating adverse effects, oral corticosteroids do not prevent airway remodeling as they have no effect on IL-33, the cytokine responsible for remodeling the airway in asthmatic patients [11].

For most patients, asthma is adequately controlled with traditional therapies. One study shows that approximately sixty-three percent of patients who initiated biologic therapy for asthma had suboptimal medication adherence to traditional therapy [10]. Despite this, approximately fifteen percent of all asthma cases remain uncontrolled, even with the adherent use of these therapies [11]. In this patient cohort, GINA and National Heart, Lung, and Blood Institute (NHLBI) recommends the addition of biologic therapy [11]. These agents can be considered if a patient’s asthma symptoms persist or worsen while receiving therapy with traditional agents, such as beta-agonists, muscarinic-antagonists, and corticosteroid supplementation, either alone or in combination [12]. Most studies have suggested a benefit in adding a biologic agent if two or more courses of oral corticosteroids are required per year for asthma exacerbations [7].

Before biologic therapy is initiated, it is important to confirm the diagnosis of asthma and measure relevant biomarkers, such as eosinophil count, IgE levels, and FeNO. These biomarkers will become increasingly important in guiding targeted biologic therapy and identifying patients most likely to respond to biologic therapy [13]. Currently, the greatest hindrance for patients who require biologic therapy is the high cost. For example, a study analyzing the cost-effective benefits of mepolizumab in patients with eosinophilic asthma found that a sixty percent reduction in price was needed to offset the quality-adjusted life years (QALY) of standard care alone [14]. However, biologics may not be needed long-term. A study evaluating the termination of biologics after six to twelve months of use in patients with controlled asthma versus those who continued therapy found similar rates of exacerbation in the following six months [15]. Another observational, multicenter study demonstrated that over sixty percent of patients who were present on the International Severe Asthma Registry were eligible for biologics and had a substantial exacerbation burden. For those patients, the initiation of biologic agents leads to a lower incidence of exacerbations [16]. Furthermore, patients who stopped biologic therapy experienced worsened clinical outcomes and more emergency healthcare resource utilization in patients with severe asthma than those who continued biologic therapy [17].

With six biologic agents currently approved for the treatment of asthma, and many in development, it is safe to assume that the utilization of these will be tailored to specific biomarkers and immunological phenotypes after the exhaustion of traditional therapies, barring a substantial change in affordability [14,18,19]. As indications for when to use biologics in the management of severe asthma expand, additional research is needed to conclusively determine the best practices of management in patients with severe asthma [20].

## 4. Various Biologic Agents

Below, follows a more in-depth discussion of the various biologic agents approved for use in the United States for patients suffering from severe asthma refractory to conventional therapy.

### 4.1. Omalizumab (Anti-IgE)

Omalizumab (Xolair) is a recombinant deoxyribonucleic acid (DNA)-derived humanized monoclonal antibody (mAb) directed against free human IgE. It was the first biologic approved for the treatment of bronchial asthma. It binds the Fc portion of IgE, leading to a reduction in free IgE levels, which in turn prevents further IgE binding to the FCεRIs. This results in the prevention of mast cell degranulation and inhibition of inflammatory mediator release [21]. Since IgE regulates its own receptors, omalizumab reduces the expression of FCεRI by reducing the free IgE [21].

In one systematic review involving twenty-five randomized control trials (RCTs), omalizumab was associated with an approximate twenty-five percent reduction in asthma exacerbations and allowed reduction of inhaled corticosteroids, when compared to the placebo [22]. In the INNOVATE study, omalizumab was used in patients with poorly controlled asthma with high-dose ICS and LABA therapy. The study results revealed significantly reduced asthma exacerbations [23]. One randomized controlled trial, the EXTRA TRIAL, demonstrated that omalizumab use was associated with a twenty-five percent reduction in asthma exacerbations [24]. Another prospective study involving 806 patients revealed that 87% of the enrolled patients showed clinical improvement in symptoms and lung function [25].

Omalizumab can be prescribed to patients above six years of age with difficult-to-control symptoms with inhaled corticosteroids, positive skin allergy testing, and high total serum IgE levels (see Table 1). In patients, 6 to 11 years of age, the total IgE levels should be between 30 and 1300 IU/mL, while for patients with ages greater than 12 years, the IgE levels need to be between 30 and 700 IU/mL. These recommendations are valid for the United States; however, in the European Union, the IgE levels should be between 30 and 1500 IU/mL [26]. In addition to traditional asthma, omalizumab has demonstrated efficacy in the treatment of an aspirin-exacerbated respiratory disease, characterized by leukotriene overproduction, severe asthma, and nasal polyposis [27]. Similarly, a clinical trial recently demonstrated the efficacy of omalizumab in the treatment of pediatric atopic dermatitis [28].

Omalizumab is administered as a subcutaneous injection every two to four weeks. Peak serum concentrations are reached after an average of seven to eight days due to the slower absorption rate of omalizumab. It can be continued indefinitely after determining an appropriate clinical response over three to six months [27]. The dosing of omalizumab is largely dependent on patient weight and pretreatment IgE levels, as the best available evidence suggests a 15:1 serum omalizumab to baseline IgE ratio is needed to adequately suppress the IgE inflammatory response [29]. Omalizumab has a 0.1% risk of anaphylaxis, essentially impacting 1 patient per 1000. It can occur at any time within twenty-four hours of the dose being administered. EXCELS, a post-marketing observational cohort study, was performed to gather safety profile data for omalizumab. This study revealed a higher incidence of cerebrovascular and cardiovascular events [30]. However, after controlling for various confounders, the reduction in the effect of an increased risk of such events was noted [30]. Current asthma management should not be affected by the findings of the EXCELS study in patients receiving omalizumab. However, healthcare professionals should be mindful of a possible association of omalizumab with cerebrovascular and cardiovascular events. There are no recommendations for the use of omalizumab during pregnancy and breastfeeding.

### 4.2. Reslizumab (Anti-IL5)

Reslizumab (Cinqair) is a biologic agent which received approval in 2016 from the Food and Drug Administration (FDA). Reslizumab has a mechanism of action that involves binding to IL-5 on eosinophils, to interfere with cell differentiation. IL-5 is known, based on prior studies, as a cytokine that promotes eosinophil growth, differentiation, recruitment, activation, and survival [31]. Clinical studies have demonstrated that patients receiving reslizumab have shown a ninety-two percent reduction in mean eosinophil counts compared with a twenty-one percent reduction in those given a placebo [32].

Reslizumab has undergone several randomized, double-blinded, placebo-controlled trials. The agent has proven to reduce exacerbation rates with relative reductions of fifty percent and forty-one percent demonstrated in two separate studies [33,34]. Another clinical trial investigated the improvement in forced expiratory volume in the first second (FEV1) in patients undergoing treatment with reslizumab. The trial found an FEV1 change of 0.160 L greater than the placebo [35]. The agent is approved for patients eighteen years of age and older as an add-on maintenance therapy for patients suffering from severe asthma with an eosinophilic phenotype [31]. In prior studies, the agent has demonstrably reduced exacerbation rates and severity of patients eighteen years and older who continue to suffer from uncontrolled severe eosinophilic asthma, despite adequate corticosteroid therapy. Reslizumab has shown improvement in lung function in asthma patients with eosinophil count >400 cells/microliter (µL), although it failed to reveal an improvement in FEV1 in patients with baseline eosinophils < 400 cells/µL [36]. An absolute eosinophil count of >400 cells/µL is a high hurdle to get over, which may limit its usefulness. Unlike other agents, prior research has determined that reslizumab is not appropriate for use with other eosinophilic conditions, acute bronchospasm, or status asthmaticus [32].

Reslizumab is administered via intravenous infusion every four weeks. Dosage is weight-based, with a typical dosage of 3 milligrams (mg)/kilograms (kg) infused over 20 to 50 min. The most serious adverse reaction to reslizumab seems to be anaphylaxis, with a 0.3% rate of patients reporting anaphylaxis. Other adverse reactions may include myalgias and transient creatine phosphokinase (CPK) level elevations [31].

### 4.3. Benralizumab (Anti-IL5)

Benralizumab (Fasenra) is an IL-5 receptor, alpha-directed cytolytic mAb that targets eosinophils for their destruction. Benralizumab acts by reducing eosinophils and basophils through antibody-dependent cell-mediated toxicity. Benralizumab has proven to be effective for asthmatics greater than 12 years of age, who have an eosinophilic predominance greater than 300 cells per microliter and do not respond to other GINA, Steps 4–5, therapies [26].

Multiple trials have established the efficacy and safety of the agent in patients with severe uncontrolled asthma, using inhaled corticosteroids and beta-agonists. In phase 3 of the SIROCCO study, which included 1204 patients, benralizumab was noted to have a 51% reduction in the annual rate of asthma exacerbations in patients with an eosinophil count greater than 300 cells per μL, at 48 weeks of treatment [37]. In the same study, benralizumab showed an improvement in FEV1 compared to the placebo [37]. Similar to this, after fifty-six weeks of benralizumab therapy for severely uncontrolled asthma patients, the CALIMA trial was able to demonstrate both a decrease in exacerbations and an improvement in lung function [38]. Another randomized, double-blinded, multicenter study investigated the effect of benralizumab on a reduction in oral glucocorticoid dose and noted a seventy-five percent reduction in daily use of oral glucocorticoid doses while maintaining asthma control. This study found four times higher odds of reducing daily glucocorticoid dosage, compared to the placebo [39].

The ZEPHYR 2 study is another recent study that demonstrated results that were consistent with a reduction in asthma exacerbation rates. The study investigated two cohorts, in which patients aged twelve or older were treated with benralizumab and monitored for up to twenty-four months. The results demonstrated that the patients had reduced rates of asthma exacerbation, thus, demonstrated robust long-term reduction, following the initiation of benralizumab [40].

The agent was administered as a 30 mg subcutaneous injection, given once every four weeks for the first three doses, followed by once every eight weeks thereafter. Headache and pharyngitis are among the most common adverse reactions to the drug seen in over five percent of patients. Other adverse reactions include injection site reactions (pain, erythema, pruritus, and papule development), which occurred at a rate of 2.2%, compared to in 1.9% of patients treated with placebo [41].

### 4.4. Mepolizumab (Anti-IL5)

Mepolizumab (Nucala) was the first anti-IL-5 mAb approved as an add-on maintenance therapy for the management of severe eosinophilic asthma. The agent prevents IL-5 interaction with IL-5Rα. This interaction takes place on the eosinophil surface and will alter bone marrow eosinophil differentiation and a decreased number of extracellular matrix proteins in the reticular basement membrane of airway mucosa [42].

Numerous small randomized controlled trials demonstrated success with mepolizumab. Mepolizumab was noted to reduce levels of sputum and blood eosinophils, along with asthma exacerbations [43,44]. Subsequently, the large phase IIb/III multicenter DREAM trial showed a significant reduction in sputum and blood eosinophil counts as well as annualized asthma exacerbation rates. Eligible participants in the DREAM trial included 621 patients aged 12 to 74 years, with a history of severe, recurrent asthma exacerbations, and an underlying eosinophilic inflammatory response [45]. Two additional randomized controlled trials supported the DREAM trial and demonstrated the efficacy of mepolizumab, namely the Steroid Reduction and Mepolizumab Study (SIRUS) and Mepolizumab as an Adjunctive Therapy in Patients with Severe Asthma (MENSA) trial. These trials demonstrated a reduction in the oral corticosteroid dosage in patients treated with mepolizumab versus placebo, decreased exacerbation rates, improved asthma symptoms, marginal increases in FEV1, and significant improvement in quality of life [45,46,47]. A subsequent analysis of the DREAM and MENSA studies demonstrated a reduction of forty-seven percent in the mean exacerbation rate [46,48].

Mepolizumab is indicated in patients aged greater than twelve years with eosinophil levels greater than or equal to 150 cells/µL within six months of dosing [47,48,49,50,51,52]. The agent is given as a 100 mg dose every four weeks by subcutaneous injection [53]. Adverse effects include hypersensitivity injection site reactions, headaches, back pain, fatigue, and herpes zoster infections [53].

### 4.5. Dupilumab (Anti-IL4/IL13)

Dupilumab (Dupixent) is a human mAb directed against the alpha subunit of the IL-4 receptor, which results in the blockage of IL-4 and IL-13, and in turn, prevents IgE production and inflammatory cell recruitment accountable for TH2 inflammation [54]. Dupilumab is particularly effective in asthma management as it antagonizes both IL-4 and IL-13 [55]. In the LIBERTY ASTHMA QUEST trial, dupilumab demonstrated a significant reduction in FeNO and other type 2 inflammation biomarkers (including IgE), confirming its biologic activity on airway inflammation [56]. Published research has demonstrated that dupilumab acts to decrease asthma exacerbations, reduce oral steroid use, improve lung function, and result in the decrease of type 2 inflammation [57]. In the VENTURE trial, dupilumab showed similar results, decreasing the rate of severe exacerbations by fifty-nine percent; half of the patients previously dependent on oral corticosteroids were able to discontinue the use of steroids following treatment with dupilumab [58]. As demonstrated by the VENTURE trial, dupilumab therapy resulted in a persistently high reduction in oral corticosteroid use without a tapering scheme of reduction for the TRANSVERSE trial [59].

Dupilumab is indicated for patients with oral corticosteroid-dependent asthma or eosinophilic phenotype. It can be given to patients above twelve years of age with moderate to severe asthma and has demonstrated improved lung function, a decrease in asthma exacerbations, and an acceptable side effect profile in the VOYAGE trial, which analyzed patients between six and eleven years of age [59]. In addition to asthma, dupilumab also has a proven efficacy in two phase-III trials, where it reduced the symptoms of atopic dermatitis, thereby improving the quality of life in affected patients [60]. While data are sparse, several randomized controlled trials showed that dupilumab had relatively similar safety profiles, compared to placebos, although it did show a higher incidence of injection site reactions and transient blood eosinophilia [56]. A subsequent analysis assessed the agent’s efficacy in comparison to a placebo in subpopulations of patients with type 2 asthma using high-dose inhaled corticosteroids. The analysis determined that the drug decreased asthma exacerbation rates, improved lung function, and allowed patients more control over their disease [61]. Dupilumab is administered as a starting dose of 600 mg and afterward is given as a dose of 300 mg every alternative week [54].

### 4.6. Tezepelumab (Anti-Thymic Stromal Lymphopoietin)

Tezepelumab (Tezspire) is a mAb specific for thymic stromal lymphopoietin (TSLP) and was recently approved for the treatment of severe asthma that cannot be controlled with traditional agents [62]. TSLP is a cytokine primarily responsible for upregulating the production of cytokines by antigen-specific TH2 cells and has been demonstrated to be present in much higher levels in patients with severe asthma compared to patients without asthma [63,64].

A correlation has been shown between the increasing severity of the disease and increasing levels of TSLP in asthma patients [65,66]. Of note, it is the first biologic approved for severe asthma independent of asthma phenotype and serum biomarker levels [67]. The upstream TSLP monoclonal antibody has been shown to reduce FeNO, IgE, and blood eosinophil count [68]. To date, only omalizumab has demonstrated reductions in all three biomarkers, albeit with less efficacy compared to tezepelumab [69].

PATHWAY, a phase II clinical trial, compared the use of tezepelumab to a placebo in 550 patients with severe asthma and demonstrated that tezepelumab is superior in preventing asthma exacerbations [62]. Exacerbation rates were lower in the tezepelumab groups by roughly sixty to seventy percent, when compared to the placebo, depending on the dose and frequency of drug delivery of tezepelumab. The optimal dose in this study to alleviate asthma exacerbations and limit adverse effects was found to be 210 mg every 4 weeks, as evidenced by a 71% reduction compared to the placebo, and less than 1% of patients discontinuing the drug at that dose due to adverse effects [62].

The phase III NAVIGATOR trial randomized 1061 patients with severe asthma to receive either the 210 mg dose of tezepelumab or a placebo every 4 weeks. Results of the trial determined that the asthma exacerbations had an annualized rate of 0.93 for the tezepelumab group and a 2.10 annualized rate for the placebo group. Pulmonary function tests improved at much greater rates in the tezepelumab group compared to the placebo group after the year-long conclusion of the trial. Frequencies of adverse effects were similar between the two, indicating that tezepelumab is superior to the placebo in improving outcomes in patients with severe asthma [70].

A post hoc analysis of the phase III NAVIGATOR trial revealed that in addition to tezepelumab promoting a reduction in asthma exacerbations per year and improvements in lung function, the trial also involved patients who were already taking additional asthma controller medications [71]. This analysis examined how patients benefited from tezepelumab, while also receiving other controller medications. There were 1059 patients, subdivided into 528 patients, receiving tezepelumab and 531 receiving a placebo, of whom 493, 381, and 185 were receiving 1, 2, or more than 3 additional controller medications, respectively [71]. The analysis found a reduction of forty-one percent in the mean exacerbation rate when treated with tezepelumab, compared to the placebo, for the patients with one additional controller subgroup, and by sixty-eight percent and sixty-one percent for the two additional and greater than three additional subgroups, respectively. In addition, there was an improved prebronchodilator FEV1 compared to the placebo across all subgroups [71].

An additional review of published data revealed that more research into the drug’s efficacy was needed. SOURCE, a phase III clinical trial, evaluated the oral corticosteroid-sparing ability of tezepelumab. The trial results indicated a failure of tezepelumab to reduce the oral corticosteroid dose at forty-eight weeks when compared to the placebo [72]. Another study, CASCADE, revealed that tezepelumab reduces mucus plugging and airway hyper-responsiveness by reducing airway eosinophil count [73].

Tezepelumab is given via subcutaneous injection at a dose of 210 mg every 4 weeks. Its fixed dose and administration schedule is favorable compared to other existing add-on therapies as it does not require a loading phase, weight-based dosing, or any dosing interval adjustment [67]. Localized injection site reactions appear to be the most common adverse reaction, while emergent adverse reactions were found to be extremely rare [74,75,76].

**Table 1:** A summary of biologic agents used in the treatment of asthma. Created by the authors.

Drug	Mechanism of Action	Route of Administration	Indication	Contraindication
Omalizumab	Binds IgE to prevent binding to high-affinity IgE receptors (FcεRI) on dendritic, basophils, and mast cells	Subcutaneous	Treatment of moderate to severe persistent allergic asthma for patients aged ≥6 years with positive skin test or in vitro reactivity to a perennial aeroallergen and uncontrolled on inhaled corticosteroids	Known hypersensitivity reaction
Reslizumab	Binds to IL-5	Intravenous	Add on maintenance treatment of patients with severe asthma aged ≥18 with an eosinophilic subtype	Known hypersensitivity reaction
Benralizumab	Binds IL-5R on eosinophils inducing natural killer cell-mediated eosinophil apoptosis	Subcutaneous	Add on maintenance treatment of patients with severe asthma aged ≥12 and with an eosinophilic phenotype	Known hypersensitivity reaction
Mepolizumab	Binds to IL-5	Subcutaneous	Add on maintenance treatment of patients with severe asthma aged ≥6 with an eosinophilic phenotype	Known hypersensitivity reaction
Dupilumab	Binds to IL-4R alpha inhibiting IL-4 and IL-13 signaling	Subcutaneous	Add on maintenance treatment of patients aged ≥6 with moderate to severe asthma characterized by an eosinophilic phenotype or with oral corticosteroid-dependent asthma	Known hypersensitivity reaction
Tezepelumab	Binds to thymic stromal lymphopoietin (TSLP)	Subcutaneous	Add on maintenance treatment of adult and pediatric patients ≥ 12 with severe asthma	Known hypersensitivity reaction

## 5. Conclusions

In this review, we have sought to compile and discuss existing evidence related to currently approved biologic therapies for asthma (Table 1). The goals of this rapidly advancing subset of therapies are improved quality of life with better control of asthma symptoms, reduction in exacerbation frequency and severity, and reduction in the use of oral corticosteroid therapy. With regard to success in achieving these goals, strong emphasis should be placed on appropriate patient selection. Available data regarding the efficacy and role of these therapies in asthma will continue to evolve and continued research is needed to ensure appropriate application and identify novel therapeutic targets.

Biologic agents for asthma have created additional options for patients who do not have adequately controlled asthma despite maximizing therapy with corticosteroids and bronchodilators. Newer agents, such as itepekimab and GSK3511294, are also in development as monotherapy or to be used in combination with other agents. With additional studies, their efficacy and safety will become clearer. Biologic therapy in bronchial asthma has opened doors for future research with regard to the availability of biomarkers to monitor treatment response, monitoring of biologic drug levels in the body, and duration of treatment, and it also warrants further research into the subject of long-term safety profile.

## Data Availability

Not applicable.
